# The Hands

**Published:** 1869-06

**Authors:** C. H. Evans


### THE HANDS.

BY C. H. EVANS.

One of the most disagreeable things connected with Dentistry since the introduction of rubber, is the handling of the blackened flasks when taken from the vulcanizer. When an operator is without an assistant, it is difficult to keep the hands in a proper condition, very necessary to the success of a Dentist. The operations are sufficiently dreaded by most persons, even when we make the manipulations as painless and agreeable as possible.

The use of brass flasks will lessen the quantity of the black deposit very much. Iron flasks seem to be the least able to resist the action of the high heat, and the sulphur contained in the rubber. It is better when the fingers are blackened, not to try to clean them by the use of pumice stone or pumice stone soap, as some endeavor to do, taking off about one half of the cuticle; but to take the sweet oil brush, and oil over the blackened spots, then rub the fingers together a few moments, which has the effect to loosen the particles. Remove the oil with castile soap, leaving the hands soft and smooth, with the advantage of having the full thickness of the cuticle; an exemption from the soreness and puckering, where the skin is worn thin.

A little good glycerine and rose water may be used with advantage after washing, especially if an operator is obliged to keep his hands in water longer than is required for perfect cleanliness.

